# Regional differences of [^18^F]-FDG uptake within the brain during fatiguing muscle contractions

**DOI:** 10.1002/brb3.319

**Published:** 2015-02-17

**Authors:** John H Kindred, Kari K Kalliokoski, Jens Bojsen-Møller, Thorsten Rudroff

**Affiliations:** 1 Department of Health and Exercise Science, Colorado State UniversityFort Collins, Colorado; 2 Turku PET Centre, University of Turku and Turku University HospitalTurku, Finland; 3 Department of Physical Performance, Norwegian School of Sport SciencesOslo, Norway

**Keywords:** [^18^F]-Fluorodeoxyglucose, performance fatigability, positron emission tomography (PET), skeletal muscle fatigue, statistical parametric mapping (SPM)

## Abstract

**Background and Purpose:**

Many studies have shown that a position task is more difficult than a force task although both are performed at a similar net muscle force. Thus, the time to task failure is consistently shown to be briefer during the position task. The contributions of the central nervous system to these two types of fatiguing contractions are not completely understood. The purpose of this pilot study was to examine differences in regional brain activity between force and position tasks using positron emission tomography (PET) with [^18^F]-Fluorodeoxyglucose (FDG).

**Methods:**

Two participants performed both a force and position task, separated by 7 days, with the elbow flexor muscles at 15% maximal voluntary contraction force. During both tasks, each participant was injected with ≈256 (SD 11) MBq of FDG. Immediately after both tasks PET imaging was performed and images were analyzed to determine FDG uptake within regions of the brain.

**Results:**

FDG uptake was greater in the occipital and temporal cortices of the brain during the position task compared to the force task.

**Conclusions:**

These findings suggest that differences in visual-spatial feedback and processing may play a role in the reduced time to failure of position tasks. Future application of these findings may lead to improved designs of rehabilitative strategies involving different types of visual feedback.

## Background and Purpose

One approach to study muscle fatigue is to compare the physiological adjustments that occur when two similar fatiguing contractions, force, and position task, are performed to failure. The force task requires the individual to sustain an isometric contraction at a given % of their maximal voluntary contraction (MVC) by pulling against a rigid restraint, whereas the position task requires the subject to maintain a constant joint angle while supporting a similar, yet more compliant load (Rudroff et al. 2010). Even though the two tasks require a similar net muscle torque during submaximal sustained contractions, the duration that the position task can be sustained is consistently briefer than that of the force task (Enoka et al. 2011). With this approach, mechanisms can be identified that limit the duration of sustained muscle contractions. Previous studies have shown that contributing factors to the briefer time to task failure of the position task include an increased rate of motor unit recruitment, the recruitment of a greater number of accessory muscles, the augmentation of reflex responsiveness, and greater rates of increase in heart rate, mean arterial pressure and ratings of perceived exertion (Enoka et al. 2011). Electromyography (EMG) is traditionally used to measure muscle activation strategies during these fatiguing contractions. EMG measures electrical activity caused by the depolarization of the muscle membrane. Depolarization, which eventually leads to muscle contractions, is initiated from the central nervous system (CNS), and is under control of both spinal and supra-spinal regions, including but not limited to the motor and frontal cortices.

Over the last decades noninvasive neuroimaging techniques have evolved to allow for the quantification of neural activity during the performance of functional tasks (Tashiro et al. 2008). One such method is positron emission tomography (PET) with the glucose analog tracer [^18^F]-Fluorodeoxyglucose (FDG). Glucose is the main energy substrate utilized by the CNS and the uptake of FDG into the brain and spinal cord has been shown to be a valid and reliable measure of CNS activity (Mishina et al. 1999; Tashiro et al. 2001). While EMG studies that give insight into the causes/contributors to decreased time to task failure during the force and position task studies are plentiful, the contributions of the CNS have not been explored sufficiently. Utilizing FDG-PET, investigations into differences in regional CNS activity can be performed and thus a possible association with reduced time to task failure can be identified. The purpose of this study was to determine if differences in CNS activity could be detected within individuals performing both a force and position fatiguing tasks. We hypothesized that there would be increased FDG uptake within the motor and frontal cortex of the brain during the more difficult position task.

## Methods

**Figure 1:**
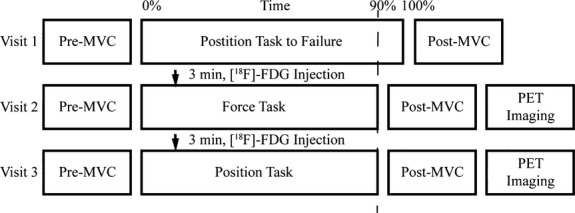
Time course for experimental procedures performed during each visit of the current study.

Two men, aged 25 and 46, without metabolic or musculoskeletal disease, consented to participate in this pilot study approved by Ethical committee of the Hospital district of South-Western Finland. The study design consisted of three visits, similar to previous studies by the authors (Rudroff et al. 2005, 2011). All experiments were performed at the Turku PET Centre, University of Turku and Turku University Hospital, Turku Finland. Visits 2 and 3 occurred after an 8 h fast, to ensure similar baseline blood glucose concentrations. During each visit participants performed isometric MVC tests before and immediately after each fatiguing task (force or position), to determine the decline in strength as an indicator of muscle fatigue. MVCs were performed for the left arm with the elbow flexed to 1.57 rad with a 3 sec increase in force from 0 to maximum effort and held for ≈3 sec. During the MVC testing strong verbal encouragement was given to the participants. The target force for each fatiguing task was performed at 15% of the measured pre-MVC. During the first visit subjects performed a position task to failure. Tasks completed during visits 2 and 3 were performed for 90% of the position task time to failure measured in visit 1 (Fig.1). Visual feedback was given during the performance of both tasks. During the force task, the elbow flexor force trace was displayed on a computer screen. Participants were required to maintain their force output at 15 ± 2.5% of their pre-MVC. For the position task an electronic goniometer was attached to the elbow and participants maintained a constant elbow angle, 1.57 ± 0.08 rad, which was visualized on a computer screen. The distance from the participants to the computer screen was similar for both tasks. Three minutes after the sustained contraction task began ≈256 (SD 11) MBq of FDG was injected into an antecubital vein on the right arm. Time from injection to imaging was approximately 22 (SD 3.9) min. After the post-MVC measurement was taken on visits 2 and 3 the participants underwent static PET imaging of the upper body. Participants performed opposite tasks at the same visit, and visits 2 and 3 were separated by 7 days. PET imaging was performed using a CTI-Siemens ECAT EXACT HR^+^ (Siemens, Knoxville, TN) PET scanner. The upper body, from the top of the cranium to the pelvic region, was scanned in 7 adjacent sections. Imaging consisted of one 5 min emission scan and 2 min transmission scans for each section. Total imaging time including transmission scans and bed transitions between sections was approximately 56 min. Figure1 displays the time course of the experimental sessions.

Utilizing Analyze 11.0 (Mayo Clinic, Rochester, MN) signal intensities for upper body PET images were converted to standardized uptake values (SUV), corrected for time of injection, body weight, and tracer dose, and cropped to allow for brain analysis by SPM8, (http://www.fil.ion.ucl.ac.uk/spm/) running on MatLab R2014a (Math Works). Images were spatially normalized and smoothed to a tracer specific template into Montreal Neurological Institute space (MNI). A paired t-test basic model approach, relative threshold set at 0.8, was utilized, with two pairs, each pair consisting of a participant's PET image taken after the performance of the force and position tasks. The *P-*value was set at 0.05 with an extent threshold set at *k *=* *250 voxels. Using given MNI coordinates from the SPM8 output areas of differing activity were labeled with corresponding areas on an automated anatomical labeling template using MRIcron (Rorden and Brett 2000).

## Results

**Figure 2:**
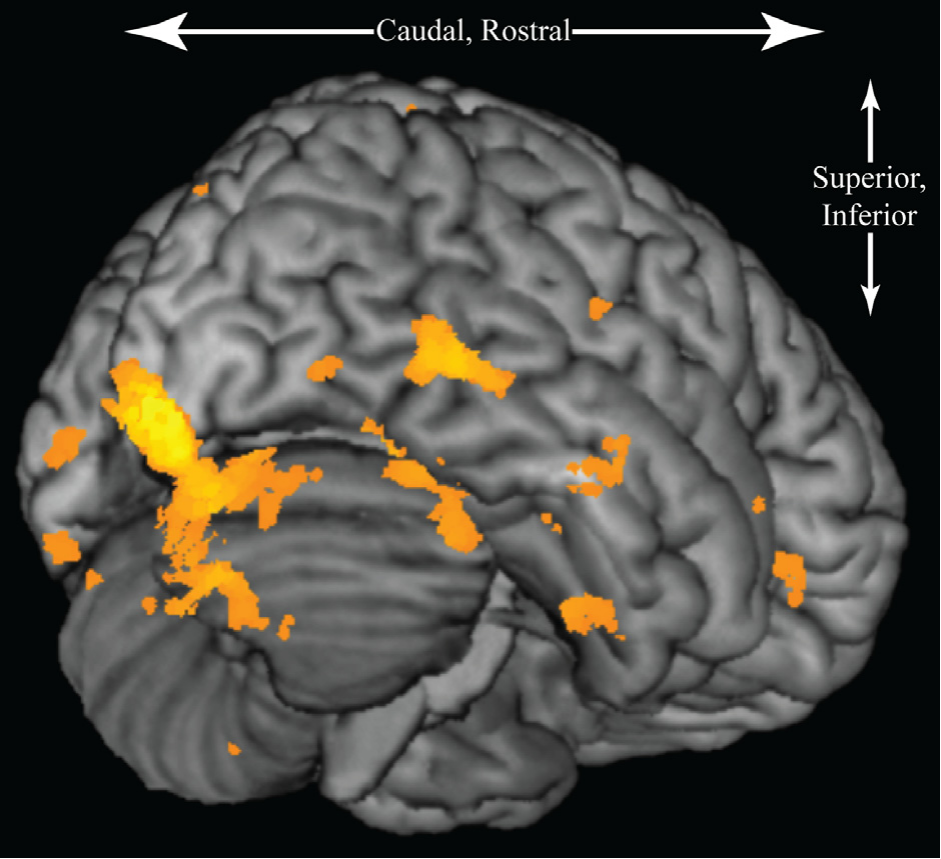
Three-dimensional rendering of the brain overlaid with regional differences in cellular activity determined by SPM8, extended threshold *k *=* *250 voxels, *P *<* *0.05. The highest activity differences are denoted in bright yellow, color threshold from 10 to 35 *T*.

MVC values and time to task failure are listed in Table 1. PET image analysis performed within SPM8 revealed several brain regions of increased FDG uptake during the position task image compared to the force task image (Fig.2). These areas include contralateral (right) areas of the occipital, frontal, and temporal cortices. No areas were found to be greater during the force task. Table 2 lists all areas and corresponding *P-*values.

**Table 1:** MVC and task times for each participant

	Subject 1	Subject 2
Time to position task failure (sec)	1080	1006
Pre-MVC (N)	304	348
Post-MVC (N)	237	265
% Decline	22	24
Time of force task	972	905
Pre-MVC	318	356
Post-MVC	245	285
% Decline	23	20
Time of position task	972	905
Pre-MVC	320	360
Post-MVC	253	295
% Decline	21	18

**Table 2:** Areas of higher [^18^F]-FDG uptake during positron-control compared to force control. Higher *T* values signify a greater difference between the position and force task. Regional coordinates expressed in MNI space (mm, mm, mm)

*T*	*P-*value	mm, mm, mm	Region	Brodmann area
35.62	0.009	18, −96, −8	Lingual_R	18
25.59	0.012	56, −50, −4	Temporal_Mid_R	21
23.03	0.014	32, −68, 38	Occipital_Mid_R	7
21.45	0.015	24, −70, 40	Occipital_Sup_R	7
21.14	0.015	40, 42, 4	Frontal_Mid_R	45
16.72	0.019	42, 36, 12	Frontal_Inf_Tri_R	45
15.19	0.021	42, 24, −10	Frontal_Inf_Orb_R	47
15.61	0.020	64, −18, −22	Temporal_Inf_R	21
14.82	0.021	−4, 28, −22	Rectus_L	11

## Discussion

The purpose of this pilot study was to determine if differences in brain activity could be detected between force and position tasks using FDG-PET. Like in previous studies the decline in muscle force was similar between the two tasks (Hunter et al. 2002; Rudroff et al. 2010, 2011) (Table 1) although differences in brain activity were detected. The results from the SPM analysis partially supported our hypothesis with increased activity in the frontal cortex in the position task, but, on the other hand, found the greatest difference between the two tasks in areas involved with integration of visual signals, including areas of the occipital and temporal cortices. Against our hypothesis, differences in motor cortex activity were not detected between tasks and activity differences in the frontal cortex were in regions mostly involved in speech and language recognition/production. This last finding could be due to verbal instructions being given to the participants during task performance.

During the performance of force and position task, previous studies have reported an increased rate of motor unit recruitment during the position task compared to the force task (Rudroff et al. 2005; Baudry et al. 2009). Movement is initiated from the motor cortex by signals that travel down the corticospinal tract to the lower motor neurons. Communication between the neurons and skeletal muscles initiate muscle contraction. The spinal cord also plays a large role in the modulation of motor signals due to the integration of signals from gamma motor neurons and Type IIb afferents. The net motor unit activity is associated with signals discharged from the spinal cord (Enoka et al. 2011). For these reasons differences in supra-spinal activity may not be significantly different between the force and position tasks because of integration performed at the level of the spinal cord (Gandevia 2001; Rudroff et al. 2005; Enoka et al. 2011) and may explain why we did not observe differences in motor cortex FDG uptake between the tasks.

The effect of visual feedback during the performance of motor tasks has been shown in multiple studies (Mottram et al. 2006; Noble et al. 2013). Mottram et al. (Mottram et al. 2006) used a similar experimental protocol, position task at 15% MVC to failure with the elbow flexor muscles, as the one used in this study but studied the effect of high and low gain feedback on the time to task failure. They showed that high gain feedback resulted in a briefer time to task failure in women but not in men. In both groups a greater decline in motor unit discharge rate was seen, but this only effected the time to task failure in women. Noble et al. (2013) used fMRI to investigate the effects of visual feedback on regional brain activity. In their protocol they tested the ability of participants to maintain 30% and 70% of maximal grip strength with and without visual feedback. Surprisingly they found that at lower force output participants were better able to match a target force without visual feedback. Both of these studies demonstrate the importance of visual feedback on task performance.

Visual feedback between force and position tasks is slightly different. Both have a representative tracing displayed on a computer monitor. Limb movement during the force task is restricted because of the attachment to a rigid restraint. However, due to the increased degrees of freedom of the position task participants are able to make a greater amount of corrections to maintain limb position. This increased movement in space could contribute to increased visual feedback. Over the last few years the sampling frequencies of the two tasks have been matched, yet there are still no published reports that measured brain activity is similar between the tasks with this change. Most of the areas determined to have higher brain activity during the position task in this study are located within the occipital and temporal cortices. Specifically the lingual gyrus, mid temporal cortex, and mid and superior occipital cortices are all strongly involved in visual-spatial processing. The ability of the participants to perceive their limb in space and the small movements that occur during task performance may lead to differences in feedback between the two tasks. This could explain the higher activity within visual-spatial integration centers within the CNS.

## Methodological Considerations

Even with a very small sample size differences were found between the two tasks. Increasing the number of subjects may reveal additional regions of more or less activity during the performance of the position task and verify the findings of this investigation. Another parameter to include in future studies is to identify differences between men and women, as Mottram et al. (Mottram et al. 2006) found sex differences with varying visual feedback in time to task failure.

## Conclusions

This study suggests CNS activity in regions involved with visual-spatial processing may play a role in the shorter time to task failure in the performance of a position task compared to a force task. These findings add to the current knowledge about force and position task performance in the literature and may contribute to refining rehabilitative strategies utilizing visual feedback in diseased populations.
